# A Novel Bimetallic Nanozyme-Driven Fluorescence–Colorimetric Dual-Mode Platform for the Rapid Detection of *Escherichia coli* O157:H7 in Meat Products

**DOI:** 10.3390/foods15173076

**Published:** 2026-08-30

**Authors:** Hongzhou Chen, Weichao Wu, Mengyu Li, Yang Liu, Chuanfu Lu, Qi Li, Yuwei Ren, Baocai Xu, Yingwang Ye, Kezhou Cai

**Affiliations:** School of Food Science and Engineering, Hefei University of Technology, Hefei 230009, China; hz.chen@163.com (H.C.); 15955177314@163.com (W.W.); mengyuli01@163.com (M.L.); orangeliuyang@163.com (Y.L.); lcf164140@163.com (C.L.); 18742033548@163.com (Q.L.); yuwei_ren2021@163.com (Y.R.); baocaixu@163.com (B.X.)

**Keywords:** bimetallic carbon-based nanozyme, *Escherichia coli* O157:H7, rapid detection, food safety

## Abstract

Foodborne diseases pose a severe threat to human health. As a critical meat-borne pathogen, *Escherichia coli* O157:H7 (*E. coli* O157:H7) can trigger severe illnesses such as hemorrhagic enteritis, threatening public safety and causing a huge economic impact on the meat industry. However, previous detection methods have struggled to strike an optimal balance among accuracy, reliability, and efficiency, with various traditional techniques exhibiting obvious technical shortcomings. To overcome these limitations, this study developed a novel biosensor for the rapid detection of *E. coli* O157:H7 based on bimetallic carbon-based nanozymes. This sensor leverages the superior peroxidase-like activity and excellent intrinsic fluorescence of bimetallic carbon dots, providing a solid foundation for constructing a dual-modal platform combining colorimetry and fluorescence. Ultimately, this collaborative approach enables the rapid and accurate detection of the pathogen, substantially enhancing the reliability and stability of the results with a limit of detection (LOD) of 10 cfu/mL. This research presents significant practical application value and offers an innovative design strategy for developing advanced diagnostic sensors for *E. coli* O157:H7 and other meat-borne pathogens.

## 1. Introduction

Meat-borne pathogens are pathogenic bacteria that cause disease or food poisoning in humans using meat products as carriers, including *Escherichia coli*, *Salmonella*, *Staphylococcus aureus*, *Listeria monocytogenes*, *Vibrio parahaemolyticus* [1,2,3]. Meat products can be contaminated with this group of bacteria during farming, processing, storage and transport [4,5,6]. Human infections can produce fever, vomiting, diarrhea, gastric colic, food poisoning, septicaemia and kidney failure [7]. *Escherichia coli* O157:H7 (*E. coli* O157:H7) is the main meat-borne pathogen belonging to the enterohaemorrhagic *Escherichia coli* (EHEC) serotypes. It is a Gram-negative bacillus without spores, with flagella and hyphae, and usually has regular cell dimensions of approximately 1.5 μm in length and 0.5 μm in width. Biologically classified in the domain Bacteria, phylum Pseudomonadota, class Gammaproteobacteria, order Enterobacterales, family Enterobacteriaceae, genus *Escherichia coli*, and species *Escherichia coli* [8], it is able to colonize the intestinal mucosa via adhesins. *E. coli* O157:H7 is one of the major pathogenic *Escherichia coli* bacteria, first isolated and named in 1982 from the feces of patients suffering from haemorrhagic colitis after eating half-cooked hamburger [9]. The bacterium can produce Shiga toxin (Stx) through its own metabolic reaction, which is its main pathogenic factor, and can inhibit the protein synthesis of host cells, leading to intestinal mucosal damage and vascular endothelial lesions, causing intestinal infections and serious complications, resulting in severe abdominal pain and bloody diarrhea [10]. As a major foodborne pathogen, this bacterium can be transmitted through food (such as unpasteurized milk, meat, fruits and vegetables, etc.) and water, and is characterized by high virulence and a low infectious dose, which can lead to human and animal infections with an exposure of only 10–100 CFU under normal circumstances [11].

Existing methods for the detection of meat-borne pathogens can be broadly classified into three categories: colony counting methods, immunological assays, and molecular biology assays [12]. The traditional colony counting method is simple and low-cost, but often time-consuming. Among the immunological assays, the immunomagnetic bead method is selective, sensitive, and specific, but it is costly and prone to affecting biological activity [13]. Enzyme immunoassays offer good specificity, high sensitivity with typical detection limits in the ng/mL range, and rapid detection that is usually completed within 2 to 4 h. However, their stability and reproducibility can be compromised by enzyme purity and environmental factors, potentially leading to false positives or false negatives [14]. Other methods also have distinct limitations, such as the high cost of protein microarrays [15] and the difficulty of accurate quantification using colloidal gold immunochromatographic strips [16]. Molecular biology detection methods include polymerase chain reaction (PCR), gene microarrays, loop-mediated isothermal amplification (LAMP), biosensors, and DNA probes. While these methods offer high accuracy, low detection limits, and rapid processing compared to traditional biochemical assays, they also face challenges such as susceptibility to contamination leading to false positives and relatively high operational costs [17]. Bimodal rapid detection technology offers unique advantages by integrating two distinct sensing modes. This integration provides a built-in cross-reference correction mechanism that effectively mitigates environmental interference. Consequently, this approach substantially enhances the accuracy and reliability of the analytical results, demonstrating broad potential for practical applications.

Natural enzymes are proteins with catalytic functions produced by living cells, possessing excellent catalytic properties and specificity. However, they are complicated to purify, costly, and unstable [18]. Carbon dots (CDs) are quasi-spherical carbon particles less than 10 nm in size that possess excellent optical properties. Their surfaces are typically enriched with functional groups such as carboxyl, amino, hydroxyl, or aromatic hydrocarbons. With increasing research, scientists have discovered that CDs also exhibit remarkable enzyme-like properties. It has been shown that carbon dot nanozymes can mimic the activities of various oxidoreductases, such as peroxidase, catalase, superoxide dismutase, and laccase [19]. In addition, the catalytic performance of carbon dot nanozymes can be further enhanced by doping metal ions or atoms (e.g., zinc, iron, copper, cobalt, manganese, etc.) into the carbon dot nanozymes, and the catalytic activity of the nanozyme can be altered by doping with different metal species or amounts to different degrees. Further, the doping of bimetallic ions or atoms can more effectively utilize the interaction between different metals to change the host–guest electronic properties and generate new catalytic sites with better nanozyme mimicry performance [20]. Nowadays, there is great interest in developing bimetallic carbon-based nanozymes and biosensors for the detection of foodborne pathogens. By integrating the intrinsic fluorescence of carbon dots with their enzyme-mimicking activity, these biosensors can achieve the rapid identification and detection of specific bacteria.

**Figure 1:**
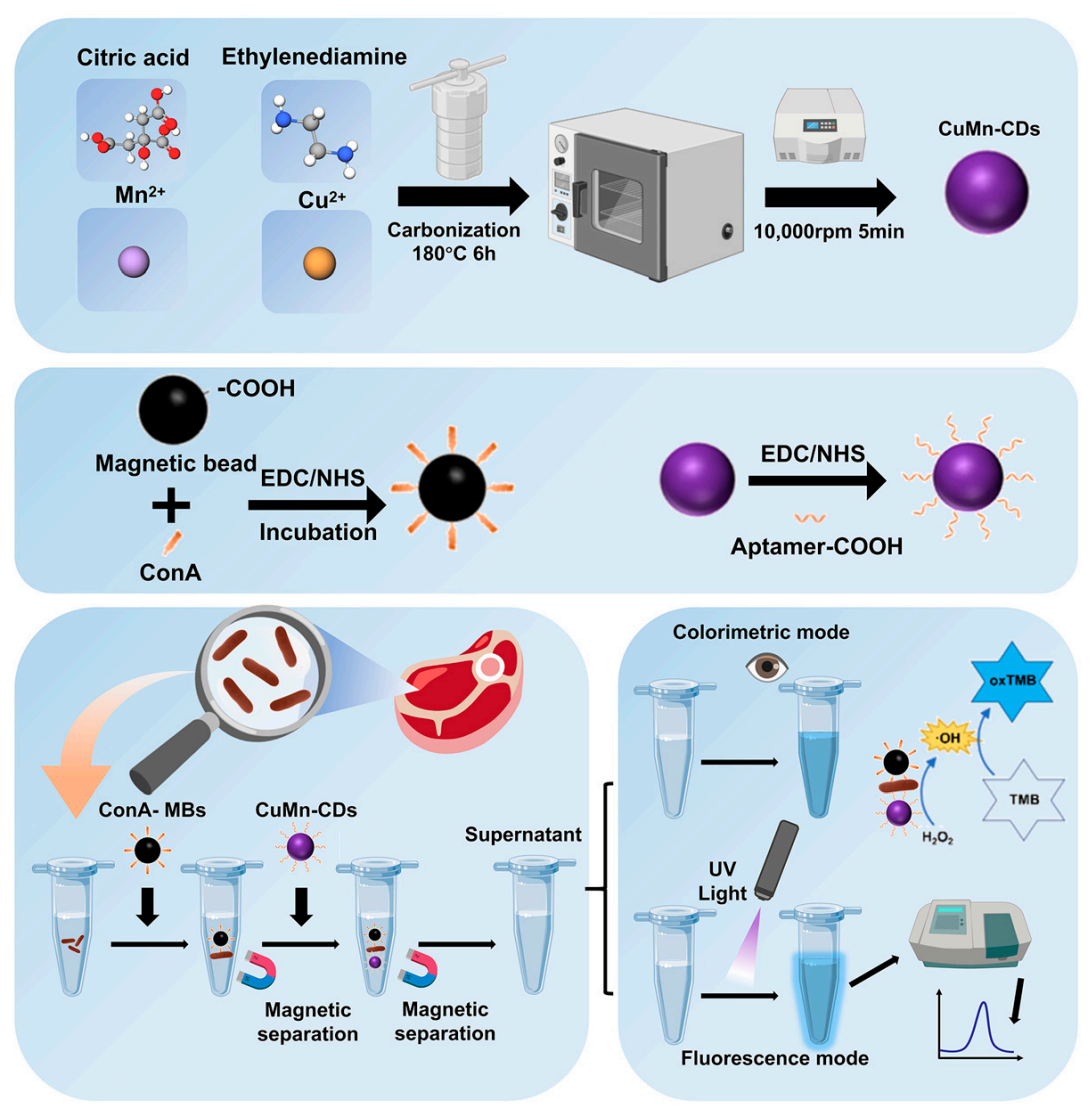
The principle of the dual-mode fluorescence–colorimetric synergistic platform for rapid screening of foodborne pathogens.

Bimetallic carbon-based nanozymes possess both oxidoreductase-like catalytic activity and intrinsic fluorescence properties. In this paper, we propose a novel bimodal rapid detection biosensor for *E. coli* O157:H7 based on these dual properties (Figure 1). The proposed biosensor relies on two distinct mechanisms: direct fluorescence signals generated upon binding to the bacteria and a colorimetric reaction in which the peroxidase-mimicking nanozymes catalyze the decomposition of hydrogen peroxide (H_2_O_2_) into hydroxyl radicals, subsequently oxidizing the colorless substrate 3,3′,5,5′-tetramethylbenzidine (TMB) into the blue product ox-TMB [21]. This study aims to investigate the feasibility of integrating these parallel signaling pathways to enhance the reliability of rapid biosensing technologies for foodborne pathogens.

## 2. Materials and Methods

### 2.1. Reagents and Materials

The specific DNA aptamer (Apt) targeting *Escherichia coli* O157:H7 was selected based on previous literature [22]. The specific sequence is as follows: 5′-COOH-CCGGA CGCTT ATGCC TTGCC ATCTA CAGAG CAGGT GTGAC GG-3′ Cat. No. [4/5406253690]. It was modified with a carboxyl group at the 5′ terminal to facilitate subsequent covalent bioconjugation, and was synthesized by Sangon Biotech Co., Ltd. (Shanghai, China); acetic acid-sodium acetate buffer, Poly (glycolic acid) (PGA) were purchased from Shanghai yuanye Bio-Technology Co., Ltd. (Shanghai, China); hydrogen peroxide (H_2_O_2_) was purchased from Sinopharm Chemical Reagent Co., Ltd. (Shanghai, China); PBS buffer solution; absolute ethyl alcohol; 3,3′,5,5′-tetramethylbenzidine (TMB, Cat. No. [CT11211-1g]) were all purchased by Beijing Coolaber Technology Co., Ltd. (Beijing, China); 1-Ethyl-3-(3-dimethylaminopropyl) carbodiimide Hydrochloride (EDC); N-Hydroxysuccinimide (NHS) were purchased by Shanghai Aladdin Bio-chem Technology Co., Ltd. (Shanghai, China); Fe_3_O_4_ magnetic beads (Fe_3_O_4_ MBs, Cat. No. [No: 3611]) were purchased from Tianjin BaseLine Co. Ltd. (Tianjin, China); concanavalin A (ConA) were purchased from Beijing Coolaber Technology Co., Ltd. (Beijing, China); copper (II) chloride dihydrate (CuCl_2_·2H_2_O), Iron (III) chloride (FeCl_3_), manganese (II) chloride (MnCl_2_) and ethylenediamine were purchased at Merck Reagents Co., Ltd. (Shanghai, China).

### 2.2. Equipment

Thermostatic culture shaker (ZHWY-103B, Shanghai Zhicheng Analytical Instrument Manufacturing Co., Ltd., Shanghai, China), CNC ultrasonic cleaner (KQ-300DA, Kunshan Ultrasonic Instruments Co., Ltd., Kunshan, China), metal bath (OSE-DB-01, Tiangen Biotech Co., Ltd., Beijing, China), biochemical incubator (SHP-80, Shanghai Jinghong Experimental Equipment Co., Ltd., Shanghai, China), ultra-clean bench (BHC-1300IIA2, Suzhou Antai Airtech Co., Ltd., Suzhou, China), high pressure steam sterilizer (DSX-280KB24, Shanghai Shenan Medical Instrument Factory, Shanghai, China), electric furnace (BDW-1200, Shanghai Shiyan Electric Furnace Co., Ltd., Shanghai, China), ultraviolet spectrophotometer (TU-1901, Beijing Purkinje General Instrument Co., Ltd., Beijing, China), fluorescence spectrometer (F97Pro, Shanghai Lengguang Technology Co., Ltd., Shanghai, China), Malvern zeta potential meter (NS-90, Zhuhai Omec Instruments Co., Ltd., Zhuhai, China), transmission electron microscope (JEM-2100, JEOL Ltd., Tokyo, Japan), ultra-low temperature refrigerator (MDF-U45V, PHC Corporation, Tokyo, Japan), general low-temperature refrigerator (BCD-610W, Haier Group, Qingdao, China), and freeze dryer (FD-1A-50, Beijing Boyikang Laboratory Instruments Co., Ltd., Beijing, China).

### 2.3. Bacterial Strains and Culture

The bacterial strains used in this study included the target pathogen *Escherichia coli* O157:H7 (ATCC 43895) and non-target interference strains: *Vibrio parahaemolyticus* (ATCC 17802), *Staphylococcus aureus* (ATCC 65389), *Cronobacter sakazakii* (ATCC 12806), *Pseudomonas aeruginosa* (ATCC 25922), *Enterococcus faecalis* (ATCC 29212), *Listeria monocytogenes* (ATCC 7644), and *Shigella flexneri* (ATCC 12022). All strains were provided by the Guangdong Microbial Culture Collection Center (GDMCC, Guangzhou, China). Prior to cultivation, the glass test tubes were wrapped, autoclaved at 121 °C for 15 min, and subsequently placed in a sterile drying oven to remove residual condensed moisture without compromising their internal sterility. For cultivation, 100 μL of all bacterial strains except V. parahaemolyticus were inoculated into 10 mL of Luria–Bertani (LB) broth and cultured in a thermostatic incubator shaker at 37 °C and 180 rpm for 8 to 10 h. V. parahaemolyticus was cultured under identical conditions in LB broth supplemented with 3% (*w*/*v*) NaCl. Following incubation, the bacterial cells were harvested by centrifugation at 1376× *g* for 5 min. To thoroughly remove residual medium components, the bacterial pellets were fully resuspended and washed three times with 5 mL of sterile PBS buffer (0.01 M, pH 7.4) per wash. The exact bacterial concentration was quantified using the standard plate counting method after incubating the agar plates at 37 °C for 24 h, and serially diluted with PBS to the desired concentrations (10^1^ to 10^7^ CFU/mL) for subsequent use.

### 2.4. Preparation of Cu-Mn CDs@Apt

#### 2.4.1. Preparation of Fe_3_O_4_ MBs

The magnetic beads (MBs) were synthesized using a facile one-pot hydrothermal method. First, 0.54 g of FeCl_3_ 6H_2_O, 1.18 g of sodium citrate, and 0.36 g of urea were dissolved in 40 mL of ultrapure water. Subsequently, 0.3 g of polyacrylamide (PAM) was added to the mixture under continuous stirring until it was completely dissolved. The resulting mixture was then transferred into a 50 mL polytetrafluoroethylene (PTFE)-lined stainless steel autoclave. The autoclave was sealed and maintained in an oven at 200 °C for 12 h. After the reaction, the autoclave was allowed to cool naturally to room temperature, and the resulting black precipitate was collected by centrifugation. The product was washed alternately with distilled water and absolute ethanol multiple times to remove impurities. Finally, the obtained Fe_3_O_4_ MBs were dried overnight in an oven at 60 °C for subsequent use.

#### 2.4.2. Preparation of MBs@ConA

First, 0.2 mL of the prepared Fe_3_O_4_ MBs suspension (with a bead concentration of 5 mg/mL, corresponding to 1 mg of MBs) was mixed with 900 μL of a 10 mM EDC and 5 mM NHS mixture prepared in ultrapure water. This mixture was stirred in a vertical stirrer for 1 h to activate the carboxyl groups on the beads. Then, 900 μL of ConA solution 1 mg/mL was added, and the reaction was carried out for 4 h in a shaker with constant rotation to synthesize the MBs@ConA complex. After the coupling reaction, unreacted ConA was removed by washing the modified MBs with acetate-sodium acetate buffer via magnetic separation. Subsequently, 1 mL of acetate-sodium acetate buffer was added to resuspend the synthesized MBs@ConA complex, yielding a final concentration of 1 mg/mL for subsequent use.

#### 2.4.3. Preparation of Cu-Mn CDs

In total, 0.05 g of citric acid, 0.07 g of CuCl_2_-2H_2_O, 0.05 g of MnCl_2,_ and 100.0 μL of ethylenediamine were dissolved in 5.0 mL of ultrapure water. After becoming homogeneous via ultrasonication, the mixture was transferred to a Teflon-lined autoclave, sealed, and placed in an oven at 180.0 °C for 6.0 h. It was removed and centrifuged twice for 15 min each to separate the supernatant. The separated supernatant was placed in a lyophilizer for 24 h under near-vacuum conditions at −50 °C. Then it was scraped out and weighed, and then 10 mL of ultrapure water was added to prepare the Cu-Mn bimetallic carbon-based nanozymes solution.

#### 2.4.4. Synthesis of Cu-Mn CDs@Apt

First, the lyophilized carboxyl-modified aptamer powder was directly dissolved in 653 μL of ultrapure water to obtain a 1 μM aptamer solution. This solution was then heated in a metal bath at 95 °C for 5 min and cooled in a refrigerator at 4 °C for 5 min to ensure proper spatial folding. Next, 160 μL of the aptamer solution was added to 250 μL of the EDC-NHS mixture and shaken for 30 min to activate the terminal carboxyl groups (-COOH) of the aptamer into amine-reactive NHS esters. Finally, 500 μL of the Cu-Mn CDs solution (which contains abundant surface amino groups, -NH_2_) was added and shaken for 2.5 h to facilitate bioconjugation via the formation of stable amide bonds.

### 2.5. Determination of the Nature of Enzyme Activity

To evaluate the enzyme-like activity, four groups of reaction mixtures (500 μL total volume each) were prepared as follows: Group 1: 50 μL of Cu-Mn@CDs solution (stock concentration: 40 μg/mL; final concentration: 4 μg/mL) + 50 μL of 10 mM H_2_O_2_ + 50 μL of 2 mM TMB + 350 μL of acetic acid-sodium acetate buffer, pH 4.5. Group 2: 50 μL of Cu-Mn@CDs solution + 50 μL of 2 mM TMB + 400 μL of acetic acid-sodium acetate buffer. Group 3: 50 μL of 10 mM H_2_O_2_ + 50 μL of 2 mM TMB + 400 μL of acetic acid-sodium acetate buffer. Group 4: 500 μL of acetic acid-sodium acetate buffer. After preparation, all mixtures were incubated in a metal bath at 40 °C for 10 min. Finally, the peak absorbance of each reaction solution at 650 nm was recorded using a UV spectrophotometer. All experiments were performed in triplicate.

### 2.6. Optimization of Cu-Mn@CDs Enzyme Activity

To systematically optimize the catalytic activity of Cu-Mn@CDs, a standard reaction system was established containing 50 μL of Cu-Mn@CDs, 50 μL of H_2_O_2_, 50 μL of TMB, and 350 μL of acetic acid-sodium acetate buffer. The mixtures were incubated for 10 min, after which the absorbance at 650 nm was measured. The specific optimization conditions were itemized as follows: pH: The buffer pH was varied from 3.0 to 7.0 (with 10 mM H_2_O_2_, 2 mM TMB, at 60 °C). Temperature: The incubation temperature was varied from 10 to 80 °C (with 10 mM H_2_O_2_, 2 mM TMB, at pH 4.5). H_2_O_2_ concentration: The H_2_O_2_ concentration was varied from 20 to 100 mM (with 2 mM TMB, at pH 4.5, 60 °C). TMB concentration: The TMB concentration was varied from 4 to 20 mM (with 10 mM H_2_O_2_, at pH 4.5, 60 °C).

Development of the enzyme-catalyzed kinetic equation: 50 μL of Cu-Mn@CDs, 50 μL of H_2_O_2_ solutions at different concentrations (20, 40, 60, 80, 100 mM), 50 μL of 2 mM TMB, and 350 μL of pH 4.5 acetic acid-sodium acetate buffer were added to the reaction mixture. The absorbance of the reaction mixture at 650 nm was continuously monitored using a UV spectrophotometer in time-course mode for 10 min at 60 °C. Next, 50 μL of Cu-Mn@CDs, 50 μL of 10 mM H_2_O_2_, 50 μL of TMB solutions at different concentrations, and 350 μL of pH 4.5 acetic acid-sodium acetate buffer were added to the reaction mixture, and the absorbance was similarly monitored continuously for 10 min at 60 °C. The initial reaction velocity (*V*) was determined by extracting the slope (ΔA/Δt) of the linear portion of the absorbance-time curve during the initial reaction phase. Subsequently, the absorbance was measured at different concentrations. The values for the Michaelis constant (*K_m_*) and the maximum velocity (*V_max_*) were obtained using the Michaelis–Menten equation provided below.

(1) $$\frac{1}{V}=\frac{K_{m}}{V_{max}[S]}+\frac{1}{V_{max}}$$

Here, *V* represents the initial reaction rate, *V_max_* represents the maximum reaction rate, *K_m_* represents the Michaelis constant, and [*S*] represents the substrate concentration.

### 2.7. Detection Procedure for E. coli O157:H7

To perform the detection of *E. coli* O157:H7, the target bacterial suspension was first mixed with ConA@Fe_3_O_4_ magnetic beads (ConA-MBs) in a sterile centrifuge tube. The mixture was incubated at 37 °C for 20 min with gentle shaking to capture the bacteria from the sample. Following this initial incubation, an external magnetic field was applied to separate the ConA-MBs-bacteria complexes, and the supernatant was discarded. After washing the complexes with PBS buffer (0.01 M, pH 7.4) to remove unbound impurities, the apt/Cu-Mn@CDs probe solution was added to the tube. This mixture was incubated for another 20 min to allow the aptamer to specifically recognize the target, thereby forming the complete ConA@Fe_3_O_4_-*E. coli* O157:H7-apt/Cu-Mn@CDs sandwich complexes. After the second incubation, a final magnetic separation was performed for 3 min. The supernatant, containing unbound apt/Cu-Mn@CDs and other matrix impurities, was carefully pipetted out and discarded. The remaining magnetic precipitate was gently washed three times with PBS buffer. Subsequently, the purified magnetic precipitate was resuspended in 200 μL of buffer. For the bimodal measurement, this resuspension was divided equally into two parts. For the colorimetric mode, TMB and H_2_O_2_ substrates were added directly to one portion of the resuspended precipitate, and the absorbance was measured at 652 nm using a UV-Vis spectrophotometer after a 10 min reaction in the dark. Simultaneously, for the fluorescence mode, the specific emission spectra of the other portion of the resuspended precipitate were recorded directly using a fluorescence spectrophotometer at an excitation wavelength of 360 nm.

### 2.8. Optimization of Optimal Excitation of Cu-Mn@CDs

Add 50 μL of Cu-Mn@CDs, 50 μL of 10 mM H_2_O_2_, 50 μL of 2 mM TMB, and 350 μL of pH 4.5 acetic acid-sodium acetate buffer, mix, and incubate at 60 °C for 10 min. Subsequently, the resulting solution was measured using a fluorescence spectrophotometer, varying the excitation wavelength (300–380 nm) and recording the resulting fluorescence intensity.

### 2.9. Conditional Optimization

Under the premise of determining the peroxidase activity of Cu-Mn bimetallic carbon-based nanozymes, conditions were optimized for lectin volume (600, 900, 1200, 1500, and 1800 μL), apt/Cu-Mn@CDs volume (20, 40, 60, 80, and 100 μL), and incubation time (10, 20, 30, 40, 50, and 60 min), respectively, in order to explore the optimal detection system for bimodal probes.

### 2.10. Sensitivity Evaluation of Bimodal Probes

First, 200 μL of *E. coli* O157:H7 (ranging from 10^1^ to 10^7^ CFU/mL), 80 μL of apt/Cu-Mn@CDs solution, and 170 μL of acetic acid-sodium acetate buffer were added to 50 μL of ConA@Fe_3_O_4_ solution. The mixture was then incubated for 40 min at 37 °C. Finally, 50 microliters of the supernatant was subjected to magnetic separation. For the colorimetric assay, 50 μL of H_2_O_2_ solution and 50 μL of TMB solution were added to 50 μL of the resuspended solution, and the reaction was maintained at 60 °C for 10 min. The absorbance of the reaction solution was then read using a UV spectrophotometer. For the fluorescence assay, another 50 μL of the resuspended solution was added to 450 μL of PBS buffer and left to stand for 10 min, and the fluorescence intensity of the reaction solution was read by a fluorescence spectrophotometer.

## 3. Results and Discussion

### 3.1. Principle

The Cu-Mn bimetallic carbon-based nanozyme from this study exhibits peroxidase-like activity. It catalyzes the decomposition of hydrogen peroxide to produce hydroxyl radicals, which, in turn, catalyze the oxidation of the colorless TMB substrate into the blue product ox-TMB [23]. This colorimetric reaction is integrated with magnetic separation technology for target detection. First, bacteria in the test sample are broadly captured by ConA@Fe_3_O_4_ via binding to their surface glycoproteins, enabling initial bacterial enrichment and magnetic separation. Next, apt/Cu-Mn@CDs are introduced to bind specifically to *E. coli* O157:H7. The aptamer serves as the main specific recognition element of the biosensor, forming a ConA@Fe_3_O_4_-*E. coli* O157:H7-apt/Cu-Mn@CDs sandwich complex only in the presence of the target pathogen. Finally, rapid recognition and detection of *E. coli* O157:H7 can be achieved through colorimetric analysis and absorbance measurements of the separated precipitates. Furthermore, this sandwich complex can also be used for the quantitative detection of *E. coli* O157:H7 by measuring specific emission peaks using a fluorescence spectrophotometer. Although generated via parallel optical pathways, these two signals function complementarily to improve analytical reliability. Originating from the exact same pathogen-capture event and Cu-Mn@CDs label, they provide a built-in cross-validation mechanism. In complex meat matrices, if one readout is compromised by background interference, the other acts as an internal reference. This functional synergy minimizes false results and seamlessly combines rapid on-site visual screening (colorimetry) with highly sensitive laboratory quantification (fluorescence).

### 3.2. Characterization of ConA@Fe_3_O_4_ and Cu-Mn@CDs

**Figure 2:**
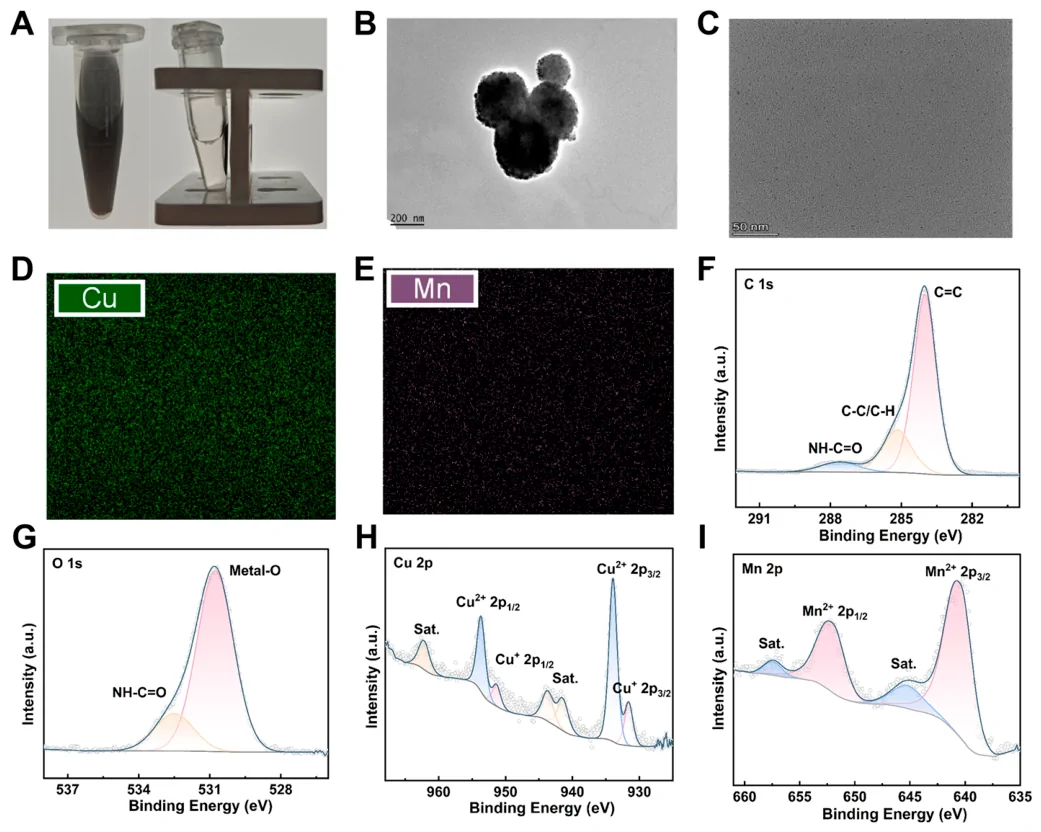
(**A**) Magnetic properties of the ConA@Fe_3_O_4_ capture probe. (**B**) TEM image of the bare Fe_3_O_4_ MBs. (**C**) TEM image of Cu-Mn@CDs (**D**,**E**) elemental mapping images of Cu-Mn@CDs; (**F**–**I**) the fitted high-resolution C 1s, O 1s, Cu 2p, and Mn 2p XPS spectra of Cu-Mn@CDs.

Following the synthesis of the MBs, ConA was conjugated to their surface to obtain the ConA@Fe_3_O_4_ complexes. The magnetic separation capability of the synthesized complexes was evaluated, and as shown in Figure 2A, the complexes exhibited effective magnetic responsiveness. Furthermore, the morphology of the synthesized magnetic beads was characterized using a transmission electron microscope (TEM), with the results presented in Figure 2B. The TEM image reveals that the bare Fe_3_O_4_ MBs possess a uniform spherical structure with a diameter of approximately 300 nm, providing a solid foundation for the subsequent surface functionalization with the lectin.

The synthesis of Cu-Mn bimetallic carbon-based nanozymes was followed by the binding of nanozymes and aptamers to synthesize Cu-Mn bimetallic carbon-based nanozymes@aptamer complexes. The morphology of the Cu-Mn@CDs was characterized using transmission electron microscopy (TEM), as presented in Figure 2C. The TEM image reveals that the synthesized bimetallic carbon dots are well-dispersed and exhibit distinct spherical nanostructures. The elemental mapping results of Cu-Mn@CDs are shown in Figure 2D,E. It can be seen that the Cu and Mn elements in the bimetallic carbon dots material are widely and uniformly distributed, which proves that the two elements are well and uniformly bonded with the carbon dots.

Figure 2F shows the XPS spectrum of C 1s, where 284.00 eV corresponds to the C=C bond, 285.16 eV corresponds to the C-C bond, and 287.63 eV corresponds to the NH-C=O bond. Figure 2G shows the XPS spectrum of O 1s, where the peak at 530.77 eV is attributed to copper-oxygen, and the peak at 532.50 eV is attributed to carbon-oxygen, with the ratio of copper to oxygen being approximately 0.2 [24]. Figure 2H shows the XPS spectrum of Cu 2p, where the peaks of Cu^+^ 2p3/2 and Cu^+^ 2p1/2 are located at 931.5 eV and 951.34 eV, respectively, and the peaks of Cu^2+^ 2p3/2 and Cu^2+^ 2p1/2 are located at 933.94 eV and 953.66 eV, respectively. It is notable that there is a 20 eV spacing between the main peaks of Cu^2+^ 2p3/2 and Cu^2+^ 2p1/2, which, according to previous research, proves the existence of CuO [25]. At the same time, there are two satellite peaks at 941.41 eV and 962.18 eV, which further proves that CuO exists in Cu-Mn@CDs [26]. Figure 2I shows the XPS spectrum of Mn 2p, where the peaks of Mn 2p3/2 and Mn 2p1/2 are located at 640.46 eV and 652.04 eV, respectively. Among them, the peak of Mn 2p3/2 is due to its simultaneous presence in the ranges of 638.84–645.83 eV and 639.93–648.72 eV, indicating the presence of Mn^3+^ and Mn^4+^ on the surface [27].

### 3.3. Probing the Enzymatic Activity Properties of Cu-Mn@CDs

**Figure 3:**
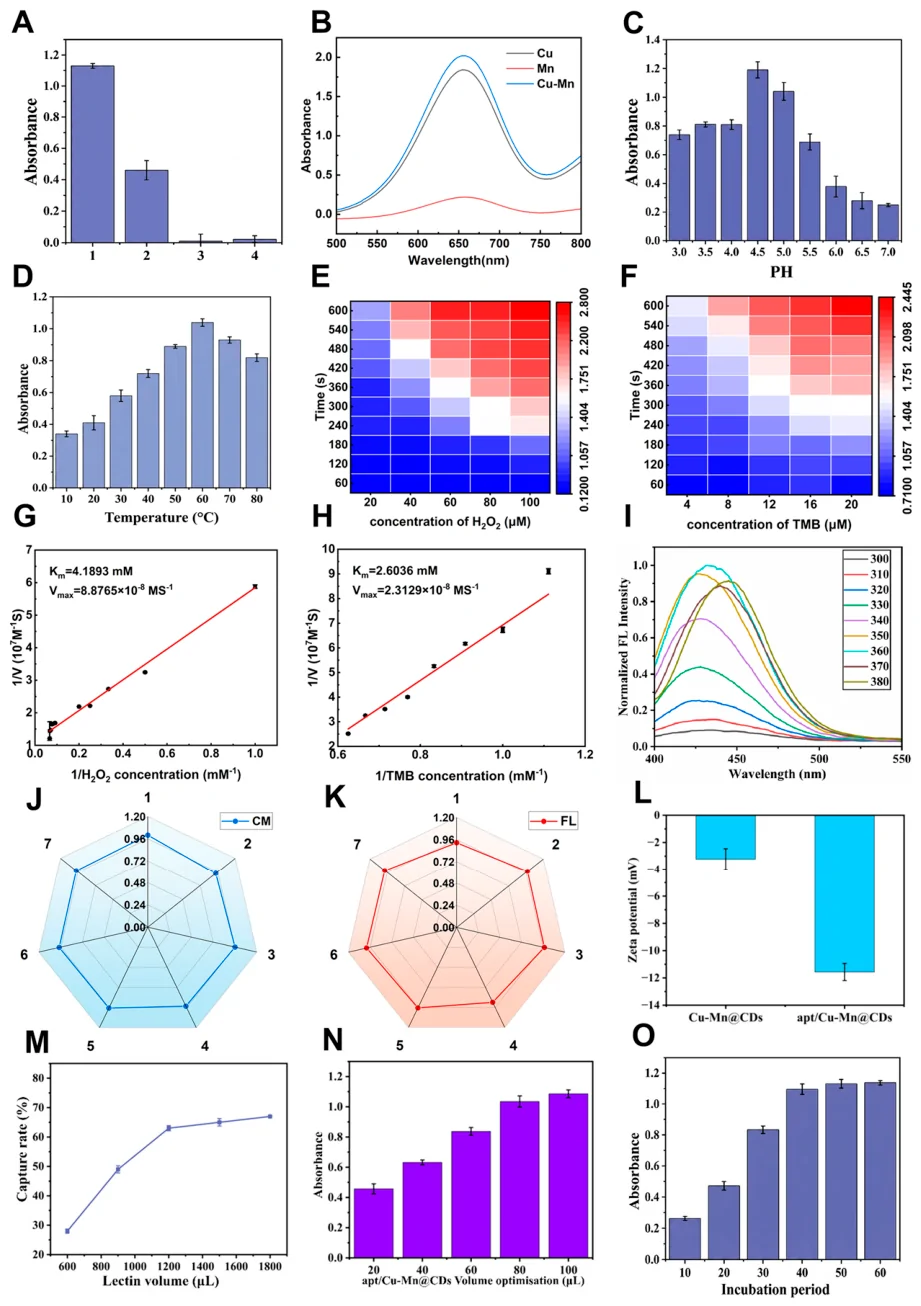
(**A**) Verification of the peroxidase-like activity of Cu-Mn@CDs in different control systems (1. Cu-Mn@CDs + H_2_O_2_ + TMB + HAc-NaAc, 2. H_2_O_2_ + TMB + HAc-NaAc, 3. H_2_O_2_ + HAc-NaAc, 4. HAc-NaAc). (**B**) Comparison of the peroxidase-like activity among undoped CDs, Cu-CDs, Mn-CDs, and Cu-Mn@CDs by measuring the absorbance of the oxidized TMB at 650 nm. (**C**) Optimization of the effect of pH. (**D**) Optimization of the effect of temperature. (**E**) Study of the concentration dependence of H_2_O_2_ in the catalytic process. (**F**) Study of the concentration dependence of TMB in the catalytic process. (**G**) Lineweaver–Burk double reciprocal plots of Cu-Mn@CDs using H_2_O_2_ as the substrate. (**H**) Lineweaver–Burk double reciprocal plots of Cu-Mn@CDs using TMB as the substrate. (**I**) Fluorescence emission spectra of Cu-Mn@CDs at various excitation wavelengths (300–380 nm). (**J**) The enzymatic activity stability of Cu-Mn@CDs over a storage period of 7 days. (**K**) The fluorescence stability of Cu-Mn@CDs over a storage period of 7 days. (**L**) Changes in the zeta potential of Cu-Mn@CDs before and after conjugation to apt. (**M**) The effect of changing lectin volume on capture efficiency. (**N**) The effect of changing apt/Cu-Mn@CDs volume on absorbance. (**O**) The effect of changing incubation time on absorbance.

The redox properties, optimal pH, and optimal temperature of Cu-Mn@CDs were explored and recorded. As shown in Figure 3A, it can be seen that Cu-Mn@CDs has the highest signal intensity in the group of 50 μL of bimetallic nano-enzymatic material + 50 μL of hydrogen peroxide + 50 μL of TMB + 350 μL of acetic acid-sodium acetate buffer, which proves that the Cu-Mn@CDs synthesized in this experiment exhibited distinct peroxidase-like activity. To verify the synergistic catalytic advantage, we compared Cu-Mn@CDs with identically synthesized mono-metallic Cu-CDs, Mn-CDs, and undoped CDs (Figure 3B). The bimetallic Cu-Mn@CDs exhibited the highest absorbance and substantially superior peroxidase-like activity compared to all controls, clearly demonstrating the enhanced catalytic performance driven by the synergistic interaction of Cu and Mn. As shown in Figure 3C, the catalytic activity at different pH levels was evaluated, and it was found that the highest signal intensity was obtained at pH 4.5, indicating that the optimal reaction pH was 4.5. Furthermore, temperature optimization was conducted while maintaining constant substrate concentrations, incubation time, and buffer pH. As shown in Figure 3D, the following eight temperature gradients (°C) were evaluated: 10, 20, 30, 40, 50, 60, 70, and 80. The average values of peak absorbance at different incubation temperatures were determined under the principle of control variables, and the highest signal intensity was finally found at an incubation temperature of 60 °C, which proved that the optimal temperature for this reaction might be 60 °C. This temperature was selected for practical assays because it strikes an optimal balance between maximizing the reaction reactivity and minimizing the energy consumption for environmental considerations.

In order to maximize the enzymatic catalytic capacity of Cu-Mn@CDs during the sensing process, we investigated its enzymatic activity. As shown in Figure 3E,F, under the condition that other factors remained unchanged, we changed different concentrations of H_2_O_2_ solutions (20–100 mM) and TMB solutions (4–20 mM). It can be clearly seen that as the concentration increased, the absorbance value also increased, indicating that the enzymatic catalytic effect was more obvious and suggesting that the nanozyme activity of Cu-Mn@CDs has a clear concentration-dependent relationship with H_2_O_2_ and TMB. To further explore its enzymatic catalytic activity, we constructed an enzymatic kinetic equation by changing the concentrations of H_2_O_2_ and TMB. As shown in Figure 3G,H, the K_m_ values of Cu-Mn@CDs were determined to be 4.1893 mM (H_2_O_2_) and 2.6036 mM (TMB) by analyzing the double reciprocal curve with K_m_ as the parameter. Its relatively low K_m_ indicates that Cu-Mn@CDs exhibits robust peroxidase-like activity. These relatively low K_m_ values are highly consistent with, and in some cases superior to, other reported peroxidase-like nanozyme systems, demonstrating that the synthesized Cu-Mn@CDs possess excellent affinity for both substrates and exhibit robust peroxidase-like activity [28].

### 3.4. Optimization of the Fluorescence Properties of Cu-Mn@CDs

As shown in Figure 3I, it can be seen that at the optimal excitation wavelength of 360 nm, the maximum emission peak is located at about 430 nm, and as the excitation light wavelength increases from 300 nm to 360 nm, the spectral intensity is enhanced in turn, which proves that the fluorescence performance of Cu-Mn@CDs is better, and its optimal excitation wavelength is 360 nm. This optimized excitation effectively maximizes the signal-to-noise ratio during sensing applications, thereby improving the sensitivity and lowering the detection limits to a highly competitive level compared with other reported carbon-based nanozyme systems. Furthermore, the long-term robustness of the material was verified. As shown in Figure 3J,K, the Cu-Mn@CDs exhibited excellent enzymatic and fluorescence stability, with no significant loss in catalytic absorbance or fluorescence intensity after prolonged storage and continuous irradiation. This exceptional stability provides a reliable material foundation for subsequent aptamer conjugation and practical sensing applications.

### 3.5. The Proof of the Successful Construction of Cu-Mn@CDs/Apts

The results of the potential measurements of Cu-Mn@CDs and apt/Cu-Mn@CDs are shown in Figure 3L, which are comparable with those of Cu-Mn @CDs, compared with Cu-Mn@CDs, the potential of apt/Cu-Mn@CDs after aptamer modification decreased, which proved that the aptamer modification was successful. This substantial shift towards a more negative potential is directly attributed to the introduction of the highly negatively charged phosphate backbone of the DNA aptamer, a trend that is highly consistent with other reported aptamer–nanoparticle conjugation studies.

### 3.6. Optimization of Sensing Conditions

#### 3.6.1. Optimization of Lectin Volume

Concanavalin A (ConA), a carbohydrate-binding lectin, was utilized in this work to broadly bind to the surface glycoproteins of bacteria. Working as a universal capture agent rather than a specific recognition element, ConA facilitates the initial magnetic capture and enrichment of the target bacteria from the sample matrix prior to the aptamer-mediated specific identification. To determine the optimal amount of this lectin for functionalizing the magnetic beads (MBs), five different volumes of ConA solution (600, 900, 1200, 1500, and 1800 μL) were evaluated for their bacterial capture efficiency. As shown in Figure 3M, the capture rate of the ConA@Fe_3_O_4_ complexes gradually increased with the addition of higher ConA volumes, reaching a maximum capture rate of approximately 68% at 1800 μL. However, beyond 1200 μL, the increase in capture efficiency slowed and stabilized, indicating that the binding capacity of the MBs for the lectin had reached saturation. This plateau occurs because all the available carboxyl functional groups on the surface of the magnetic beads are fully occupied by ConA molecules, preventing further immobilization. Therefore, 1200 μL/mg of MBs was selected as the optimal lectin volume for subsequent experiments.

#### 3.6.2. Optimal Apt/Cu-Mn@CDs Volume Optimization

The following five gradients of apt/Cu-Mn@CDs volume (μL) were set: 20, 40, 60, 80, 100. While all other conditions were kept constant, the absorbance at 650 nm was measured at different volumes, and the results were recorded as in Figure 3N, which shows that the signal intensity was gradually enhanced with the increase in apt/Cu-Mn@CDs volume, but after 80 μL, the signal intensity growth slowed down significantly; i.e., the optimal volume of apt/Cu-Mn@CDs was 80 μL. This saturation effect clearly reflects the dynamic balance between the probe concentration and the available bacterial binding sites. Once all specific antigenic sites on the surface of the captured *E. coli* O157:H7 are fully occupied by the aptamer probes, any additional probe cannot bind and is washed away, leading to a plateau in the signal.

#### 3.6.3. Optimization of Incubation Time

The following six incubation time gradients (min) were set: 10, 20, 30, 40, 50, 60. All other conditions were kept constant, and the absorbance of the reaction solution at different incubation times was measured at 650 nm; the results are shown in Figure 3O. The increase in absorbance was obvious from 10 min to 30 min, but slowed from 40 min onward; therefore, the optimal incubation time was determined to be 40 min. This 40 min duration reflects the time required for the aptamer and the target bacteria to reach a thermodynamic binding equilibrium. It represents an optimal trade-off between sensitivity and assay turnaround time, ensuring maximum target capture for high sensitivity without unnecessarily prolonging the detection process. Furthermore, all these optimized volume and time conditions are highly consistent with typical lectin-based and aptamer-based capture assays reported in the recent literature.

### 3.7. Time Optimization for Bimodal Biosensors

**Figure 4:**
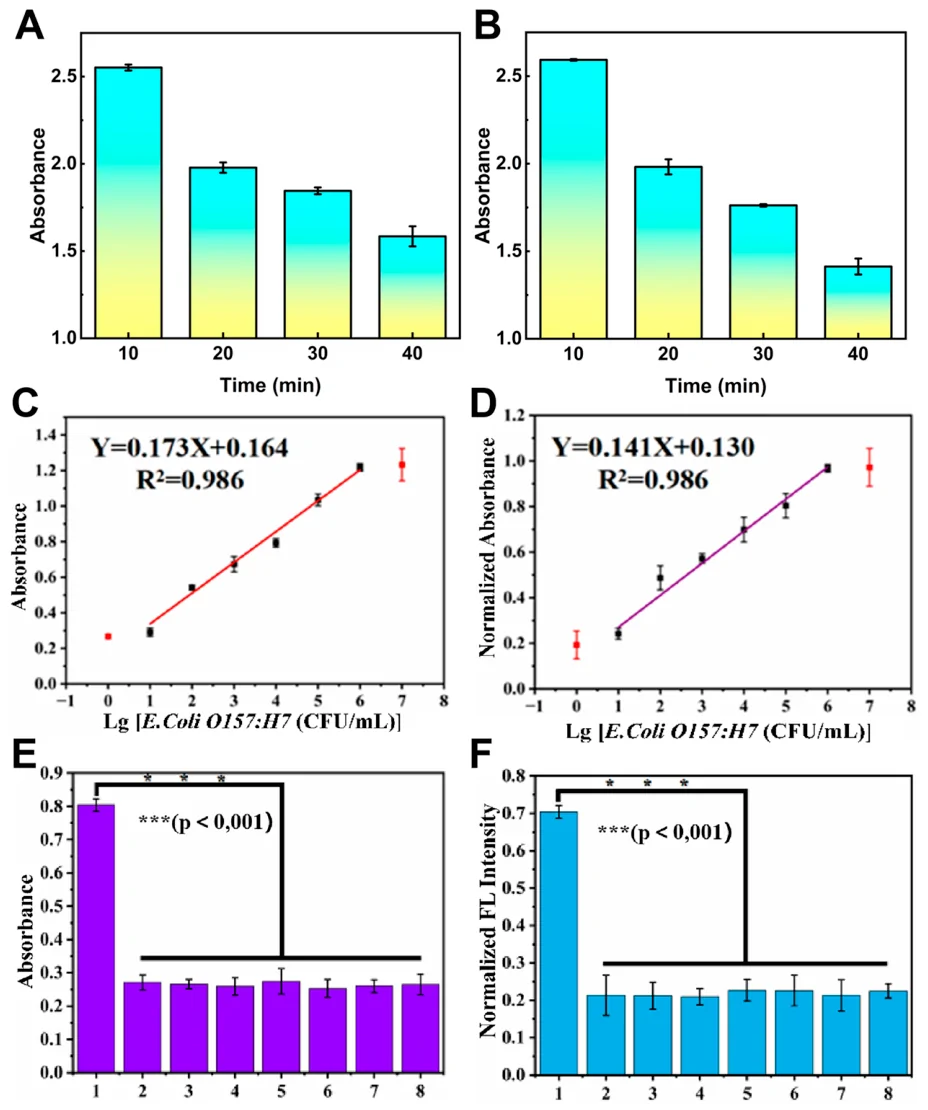
(**A**) Effect of binding time between the nanozyme and bacteria on the absorbance. (**B**) Effect of magnetic capture time on the absorbance. Calibration plots of the dual-mode biosensor in (**C**) the colorimetric mode and (**D**) the fluorescence mode. Specificity evaluation of the biosensor in (**E**) the colorimetric mode and (**F**) the fluorescence mode. The numbers from 1 to 8 represent the following: 1: *E. coli* O157:H7 (ATCC 43895); 2: *Vibrio parahaemolyticus* (ATCC 17802); 3: *Staphylococcus aureus* (ATCC 65389); 4: *Cronobacter sakazakii* (ATCC 12806); 5: *Pseudomonas aeruginosa* (ATCC 25922); 6: *Enterococcus faecalis* (ATCC 29212); 7: *Listeria monocytogenes* (ATCC 7644); 8: *Shigella flexneri* (ATCC 12022).

Before evaluating the performance of the bimodal sensor, we first investigated the specific binding of the nanozyme to the target bacterial population and the magnetic separation and screening process during the detection procedure. We first investigated the effect of binding time (10, 20, 30, and 40 min) on the specific interaction and subsequent catalysis between the nanozyme and *E. coli*, evaluating the results by monitoring the changes in absorbance. As shown in Figure 4A, as the binding time increased, the absorbance of the residual unbound bacteria obtained after magnetic separation decreased, indicating the successful construction of the sensor and demonstrating clear effectiveness by 40 min. This occurs because extended binding promotes thorough aptamer–bacteria interactions, while longer magnetic capture enhances the separation efficiency of target complexes. Therefore, 40 min is selected as the optimal assay time. This timeframe is highly competitive for real-world biosensing; it is significantly faster than traditional methods and comparable to recently reported nanozyme-based sensors, facilitating rapid food safety monitoring. Second, we investigated the magnetic capture time. As shown in Figure 4B, the absorbance decreased with increasing capture time. This is because a longer capture time increased the number of bacteria bound to the material, thereby reducing the bacterial count in the supernatant after separation and resulting in lower absorbance. Similarly, significant results were achieved at 40 min.

### 3.8. Investigation of the Sensitivity of Cu-Mn@CDs Detection Probes

The linear equations for the colorimetric and fluorescence detection modes were established, respectively. The absorbance of the reaction solution at 650 nm was measured by a UV spectrophotometer to investigate the linear relationship between different bacterial concentrations and absorbance, and the results were shown in Figure 4C. The linear regression equation was absorbance Y = 0.173 × lg [*E. coli* O157:H7 (cfu/mL)] + 0.164 (Y represents the normalized colorimetric intensity). Because the calibration curve is based on logarithmic concentrations, the exact limit of detection (LOD) was calculated using the equation LOD = 10^3σ/S^ (where σ is the standard deviation of 10 independent blank measurements and S is the slope of the curve). Based on this, the theoretical LOD was determined to be 8.6 CFU/mL. Considering that bacterial concentrations are analyzed on a logarithmic scale, this detection capability is practically reported at the order of magnitude of 10^1^ CFU/mL (i.e., 10 CFU/mL), which also represents the lowest validated concentration in our linear range. Furthermore, the fluorescence intensity of the reaction solutions was measured at an excitation wavelength of 360 nm using a fluorescence spectrophotometer. As shown in Figure 4D, a strong linear relationship was observed between the logarithm of the bacterial concentration and the fluorescence intensity. The corresponding linear regression equation was formulated as Y = 0.141 × lg [*E. coli* O157:H7 (cfu/mL)] + 0.130 (Y represents the fluorescence intensity), using the same statistical calculation method (LOD = 10^3σ/S^) mentioned above (ten batches of blank samples), the exact theoretical LOD for the fluorescence mode was determined to be 3.2 CFU/mL. Consistent with the colorimetric mode, this LOD is conservatively expressed at the 10^1^ CFU/mL order of magnitude (10 CFU/mL) for practical evaluation.

### 3.9. Specificity Assessment of Bimetallic Nanoenzyme-Based Detection Probes

Several common foodborne pathogens, which are closely related to *E. coli* O157:H7 and possess similar antigenic sites, were selected to evaluate the specificity of the assays. The detection results are documented in Figure 4E,F. As is evident from these results, the bimetallic nanozyme-based probes exhibit excellent specificity in both the colorimetric and fluorescent modes. It is worth noting that while cross-reactivity against closely related non-O157 *E. coli* strains was not directly evaluated in this study due to strain availability, the core specificity of our sandwich-type biosensor is strictly governed by the selected DNA aptamer. The exceptional target fidelity of complete biosensor systems utilizing this exact aptamer sequence against various non-O157 and non-pathogenic *E. coli* strains (e.g., *E. coli* ATCC 8739, ATCC 8798, ATCC 18683, ATCC 18684, ATCC 18685, ATCC 25922, ATCC 43895, CMCC 44102, CMCC 44825, CMCC 44829, and CMCC 44151) has been thoroughly validated in recent studies [22,29,30,31]. Since the signal output of our dual-mode platform relies entirely on specific recognition by this identical aptamer, the complete system inherently inherits this well-established specificity, effectively preventing false-positive cross-reactivity.

### 3.10. Evaluation of Actual Samples

Finally, to evaluate the broad applicability of the biosensor in the meat industry, commercial ham sausage was strategically selected as a representative model for complex meat products due to its dense matrix rich in proteins and lipids. Prior to the assay, the original ham samples were determined to be free of target bacteria by standard culture methods. For sample preparation, 25 mL of PBS solution was added to 25 g of ham and homogenized for 2 min using a homogenizer. To remove the large insoluble meat debris, the mixture was then centrifuged at 5000× *g* for 5 min. The extracted supernatant was carefully collected and manually spiked with three different concentrations of *E. coli* O157:H7 (3.5 × 10^2^, 3.5 × 10^4^, 3.5 × 10^6^ CFU/mL). These spiked samples were then quantitatively analyzed using the developed dual-mode biosensor. The analytical results are summarized in Table 1. The quantitative recovery achieved in this challenging matrix indicates the sensor’s favorable anti-interference capability and its potential for on-site pathogen screening. Although the standard plate counting method was mainly utilized to confirm the initial negativity of the sample matrix in the current stage, the acceptable recovery rates highlight the sensor’s applicability. To further advance the industry standardization of this dual-mode platform, systematic parallel comparisons with standard methods across a wider diversity of food matrices will be explored in subsequent application studies.

## 4. Conclusions

*E. coli* O157:H7 is an important pathogen that causes foodborne diseases. In summary, a novel dual-mode rapid detection biosensor for *E. coli* O157:H7 was successfully constructed based on the peroxidase-like activity and intrinsic fluorescence of bimetallic carbon-based nanozymes. This platform enables both visual and highly sensitive quantitative detection of the target pathogen in complex samples. Specifically, the colorimetric mode produces a distinct color change visible to the naked eye for rapid on-site screening without requiring complex instrumentation. Simultaneously, the fluorescence mode achieves reliable quantitative analysis using a spectrophotometer. Both modes exhibit an excellent limit of detection of 10 cfu/mL. On this basis, it provides a viable strategy for the rapid test of *E. coli* O157:H7, presenting promising application potential in the field of instant detection, which will be further strengthened by future systematic comparative validations against established standard methods.

## Figures and Tables

**Table 1:** Bimodal real-sample detection and analysis table.

Spiked Concentration (cfu/mL)	Measured (CFU/mL)		Recovery (%, 3 Batches)		R.S.D. (%, 3 Batches)	
	Abs	Flu	Abs	Flu	Abs	Flu
3.5 × 10^2^	3.3 × 10^2^	3.1 × 10^2^	94	89	17.5	15.9
3.5 × 10^4^	3.8 × 10^4^	3.4 × 10^4^	109	97	14.3	15.1
3.5 × 10^6^	3.5 × 10^6^	3.2 × 10^6^	100	91	13.6	13.9

## Data Availability

The original contributions presented in this study are included in the article. Further inquiries can be directed to the corresponding authors.

## Abbreviations

The following abbreviations are used in this manuscript:

*E. coli*	*Escherichia coli*
TMB	3,3′,5,5′-tetramethylbenzidine
CDs	Carbon dots
H_2_O_2_	Hydrogen peroxide
NHS	N-Hydroxysuccinimide
EDC	N-(3-dimethylaminopropyl)-ethyl carbodiimide hydrochloride
PGA	Poly(glycolic acid)
ConA	Concanavalin A
MBs	Magnetic beads
Apt	Aptamer
HRTEM	High-resolution transmission electron microscopy
XPS	X-ray photoelectron spectroscopy
LOD	Limit of detection
POD	Peroxidase
